# Acute Exercise Intensity and Memory Function: Evaluation of the Transient Hypofrontality Hypothesis

**DOI:** 10.3390/medicina55080445

**Published:** 2019-08-07

**Authors:** Paul D. Loprinzi, Sierra Day, Raymond Deming

**Affiliations:** Exercise & Memory Laboratory, Department of Health, Exercise Science and Recreation Management, The University of Mississippi, University, MS 38677, USA

**Keywords:** cognition, hippocampus, physical activity, prefrontal cortex

## Abstract

*Background and Objective:* The transient hypofrontality hypothesis predicts that memory function will be impaired during high-intensity exercise, as a result of a need for metabolic and cognitive resources to be allocated toward sustaining movement, as opposed to performing a cognitive task. The purpose of these experiments was to evaluate this transient hypofrontality hypothesis. *Materials and Methods:* Experiment 1 involved participants (*n* = 24; M_age_ = 21.9 years) completing four counterbalanced visits. Two visits evaluated working memory function, either at rest or during a high-intensity bout of acute exercise. The other two visits evaluated episodic memory function, either at rest or during a high-intensity bout of acute exercise. Experiment 2 (*n* = 24; M_age_ = 20.9 years) extended Experiment 1 by evaluating memory function (working memory) across 4 counterbalanced conditions, including at rest and during light (30% of heart rate reserve; HRR), moderate (50% HRR) and high-intensity (80% HRR) acute exercise. *Results:* Experiment 1 demonstrated that, when compared to rest, both working memory and episodic memory were impaired during high-intensity acute exercise. Experiment 2 replicated this effect, but then also showed that, unlike high-intensity acute exercise, memory function was not impaired during low- and moderate-intensity acute exercise. *Conclusions:* Our experiments provide support for the transient hypofrontality hypothesis. Both working memory and episodic memory are impaired during high-intensity acute exercise. Working memory does not appear to be impaired during lower exercise intensities.

## 1. Introduction

Episodic memory function refers to the retrospective recall of information from a spatial–temporal context, whereas working memory refers to the transient use of information to execute a behavior while concurrently processing conflicting stimuli [1,2]. Working memory capacity is thought to be heavily dependent on the prefrontal cortex [3], whereas episodic memory function relies on medial temporal lobe structures, such as the hippocampus [4]. Importantly, though, the prefrontal cortex and hippocampus likely interact to also influence episodic memory function [5].

As we have demonstrated experimentally, high-intensity acute exercise can enhance both episodic and working memory capacity [6,7,8]. Critically, however, the timing of this acute exercise plays an important role in memory function [9,10]. Memory function may be enhanced if the acute bout of exercise occurs prior to the memory task, whereas if it occurs during the memory task, memory performance is likely to be reduced [9,10].

This latter point may be a result of the transient hypofrontality hypothesis [11,12]. That is, when memory encoding occurs during exercise, at higher intensities, prefrontal cortex function may be compromised given that more metabolic and cognitive resources may be allocated toward sustaining movement. Although global cerebral blood flow is maintained during exercise, regional redistributions occur [13]. This transient hypofrontality hypothesis specifically theorizes that prefrontal cortex-dependent tasks (e.g., working memory tasks) may be compromised during high-intensity exercise.

The prefrontal cortex provides the infrastructure to compute complex cognitions. However, the field of cognitive psychology has shown that humans have limited information processing capacity, particularly with respect to attentional resources. Per the transient hypofrontality hypothesis, widespread neural activation of motor and sensory systems during exercise comes at the expense of prefrontal-dependent higher-order cognitions, such as memory function [11,12]. Empirical work in rodents and humans support this by showing increased neural activity in prefrontal regions involved in the control of movement, with concomitant decreases in neural activity in non-motor regions of the prefrontal cortex [14,15,16]. Moreover, in rodents, an acute bout of high-intensity exercise for 30-min has been shown to increase brain activity in many brain structures, except for select brain structures, such as the prefrontal cortex and CA3 subfield of the hippocampus [15]. Relatedly, in humans, prefrontal-dependent memory performance has been shown to be reduced during exercise, whereas cognitions requiring little prefrontal activity is unaffected [11].

The aim of the present investigation was to further evaluate the transient hypofrontality paradigm in the context of exercise. In Experiment 1, we evaluated two separate memory systems (working memory and episodic memory) to determine the robustness of the transient hypofrontality paradigm (i.e., does high-intensity exercise impair both working memory and episodic memory?). Results from Experiment 1 showed that, in alignment with this paradigm, both working memory capacity and episodic memory function were reduced during acute high-intensity exercise. As a follow-up, Experiment 2 evaluated whether this effect was intensity-dependent. Results from Experiment 2 suggest that memory function is reduced more so during high-intensity acute exercise, as compared to low- or moderate-intensity acute exercise.

## 2. Methods—Experiment 1

### 2.1. Study Design

A within-subject randomized controlled intervention was employed. Participants completed four visits, in a counterbalanced order. Two visits involved exercise, while two involved control scenarios. Specifically, participants completed an exercise visit involving a working memory assessment during exercise; a control visit also assessing working memory; an exercise visit involving an episodic memory assessment during exercise; and a control visit also assessing episodic memory. All visits occurred around the same time of day and within 48–72 h of each other. Participants provided written consent prior to participation. This study was approved by the ethics committee at the University of Mississippi (#19-043, approved on 12-18-18).

### 2.2. General Protocol for Visits

Details for the four visits are as follows (Table 1).

### 2.3. Participants

The study included 24 participants. Recruitment occurred via a convenience-based, non-probability sampling approach (classroom announcement and word-of-mouth). Participants included undergraduate and graduate students between the ages of 18 and 40 yrs. Additionally, participants were excluded if they:

Self-reported as a daily smoker [17,18]
Self-reported being pregnant [19]
Exercised within 5 h of testing [20]
Consumed caffeine within 3 h of testing [21]
Had a concussion or head trauma within the past 30 days [22]
Took marijuana or other illegal drugs within the past 30 days [23]
Were considered a daily alcohol user (>30 drinks/month for women; >60 drinks/month for men) [24]

### 2.4. Exercise Assessment

For the two exercise visits, participants engaged in treadmill exercise at 70% of their heart rate reserve (HRR).

The equation for HRR that was utilized is:

HRR = [(HR_max_ − HR_rest_) * % intensity] + HR_rest_

Heart rest (HR_rest_) was determined from the average of two resting heart rate measurements (after 5 and 6 min of seated rest) using a Polar (F1) heart rate monitor. Heart rate max (HR_max_) was estimated from the average of four heart rate max equations: Astrand et al. [25] 216.6 − (0.84 × age); Tanaka, Monahan, & Seals [26] 208 − (0.7 × age); Gellish et al. [27] 207 – (0.7 × age); and Gulati et al. [28] 206 − (0.88 × age).

### 2.5. Memory Assessment

***Episodic Memory***. For the episodic memory assessment, participants completed a paired associate learning task, as this task of cued recall is considered to be hippocampal-dependent [29,30]. Ten word pairs (two-syllable) from the Medical Research Council Psycholinguistic Database were used, with each word having an imageability score between 414–486, to reduce variability on this parameter, which can influence cue-recall performance [31]. Participants listened to each word pair via headphones, with a 5-s pause between the presentation of each word pair. After the 10th word pair was presented, participants verbally completed several simple arithmetic problems for 30-s. After this 30-s distraction period, an immediate cued recall test was performed. For this, the first word from each pair was verbally presented and the participant attempted to recall the second word from the pair. Both a short-term and long-term cued recall assessment occurred. Between the short-term and long-term assessments, participants completed simple arithmetic problems (verbally responded with the answer). Separate paired associate learning tasks occurred for the exercise and control visits.

***Working Memory***. The Brown–Peterson task was employed to assess working memory capacity. In the Brown–Peterson Task, the participant is presented with three letters at a rate of one letter per second. Following this is a series of delays where the participant is immediately given a two or three-digit random number from which the subject is asked to count backwards from, out-loud, by threes. After this, they then recall the letters that were presented prior to this arithmetic task.

For example, the subject would be presented with the letters “X C P” followed by the number “75”. The subject would have 18 s to countdown by threes from 75. Once the 18 s has passed, the subject would be asked to recall the letters that had been presented prior to the countdown.

Five trials are given for each delay period, with the delay periods including 0, 9, 18 and 36 s. All five trials with 0 s of delay are presented first, followed by a random order for each of the five trials for the 9, 18, and 36 s delay. The dependent measure is the total number of letters that was correctly recalled at each of the delay intervals. The maximum score for each interval delay is 15.

This test has demonstrated adequate test–retest reliability by Struss et al. [32,33] in both healthy subjects and individuals who had suffered a head injury. Providing evidence of sensitivity to change of the Brown–Peterson task, Coles and Tomporowski [34] demonstrated that there was a significant main effect for time following pre- versus post-exercise (F_1, 17_ = 11.36; *p* < 0.01; n^2^ = 0.40). The Brown–Peterson task has been shown to provide evidence of validity in a study by Anil et al. [35] in normal individuals where the task was positively correlated with Digit Span Backward scores at each of the delay intervals (r = 0.54 to 0.57).

### 2.6. Statistical Analysis

All statistical analyses were computed in JASP (v. 0.9.2.0). For both memory outcomes, a two-factor repeated-measures ANOVA was computed. For the working memory assessment, a 2 (conditions; exercise vs. control) × 4 (delay period of 0, 9, 18, 36 s) repeated-measures ANOVA was computed. For the episodic memory task, a 2 (condition; exercise vs. control) × 2 (time; short-term vs. long-term) repeated measures ANOVA was employed. Statistical significance was set at an alpha of 0.05. Partial-eta squared (η^2^_p_) was calculated as an effect size estimate for the ANOVA models, whereas Cohen’s *d* was calculated as an effect size estimate for the post-hoc analyses.

## 3. Results—Experiment 1

Table 2 displays the characteristics of the sample. Participants, on average, were 21.9 years of age and were predominately female (70.8%) and non-Hispanic white (95.8%).

Table 3 displays the physiological (heart rate) response to the exercise and control stimuli. For the working memory visits, there was a significant main effect for time (rest vs. endpoint), *F*(1, 22) = 280.2, *p* < 0.001, η^2^_p_ = 0.93, condition (control vs. exercise), *F*(1, 22) = 290.5, *p* < 0.001, η^2^_p_ = 0.93, and time by condition interaction, *F*(1, 22) = 1008.2, *p* < 0.001, η^2^_p_ = 0.98. Similarly, for the episodic memory visits, there was a significant main effect for time (rest vs. endpoint), *F*(1, 22) = 475.1, *p* < 0.001, η^2^_p_ = 0.96, condition (control vs. exercise), *F*(1, 22) = 443.8, *p* < 0.001, η^2^_p_ = 0.96, and time by condition interaction, *F*(1, 22) = 493.1, *p* < 0.001, η^2^_p_ = 0.96.

Table 4 Displays the memory results.

### 3.1. Working Memory

**Figure 1:**
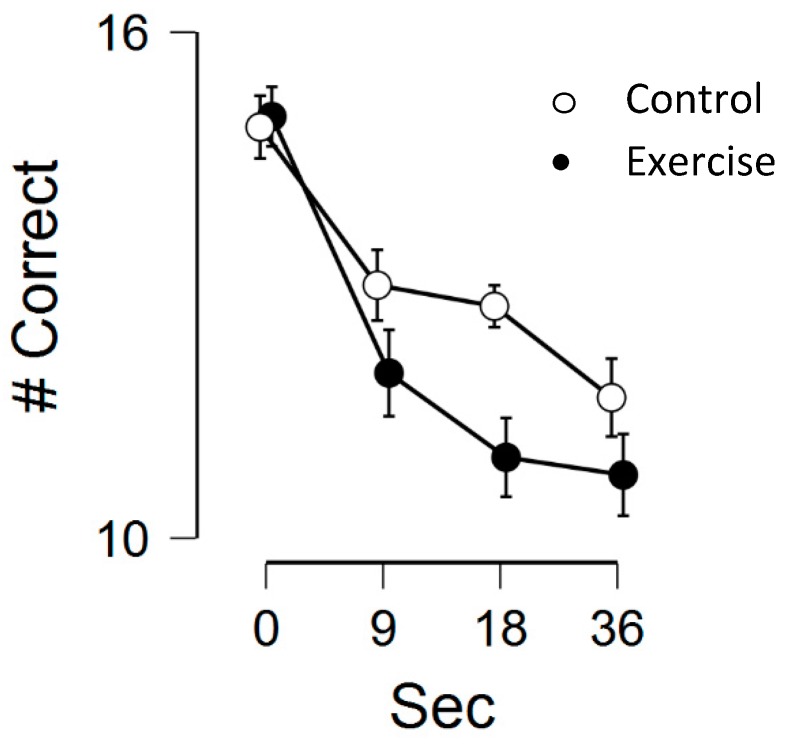
Working memory performance across the two conditions (exercise vs. control) and four time-points (0-sec, 9-sec, 18-sec, and 36-sec). Error bars represent standard errors.

There was a statistically significant main effect for time, *F*(3, 69) = 24.16, *p* < 0.001, η^2^_p_ = 0.51, main effect for condition, *F*(1, 23) = 7.59, *p* = 0.01, η^2^_p_ = 0.25, and a marginally significant time by condition interaction, *F*(3, 69) = 2.51, *p* = 0.06, η^2^_p_ = 0.10. Regarding condition effects, Bonferroni-corrected post-hoc tests indicated that the control group had higher working memory than the exercise condition, *M*_diff_ = 0.91, *p* = 0.01, d = 0.56. Regarding time effects, Bonferroni-corrected post-hoc tests indicated that the 0-sec condition had greater working memory than the 9-sec period, *M*_diff_ = 2.45, *p* < 0.001, d = 1.13, 18-sec period, *M*_diff_ = 3.08, *p* < 0.001, d = 1.25, and 36-sec period, *M*_diff_ = 3.73, *p* < 0.001, d = 1.34. There were no significant differences among other combinations. See Figure 1 below for the working memory scores across the two conditions and four time-points.

### 3.2. Episodic Memory

**Figure 2:**
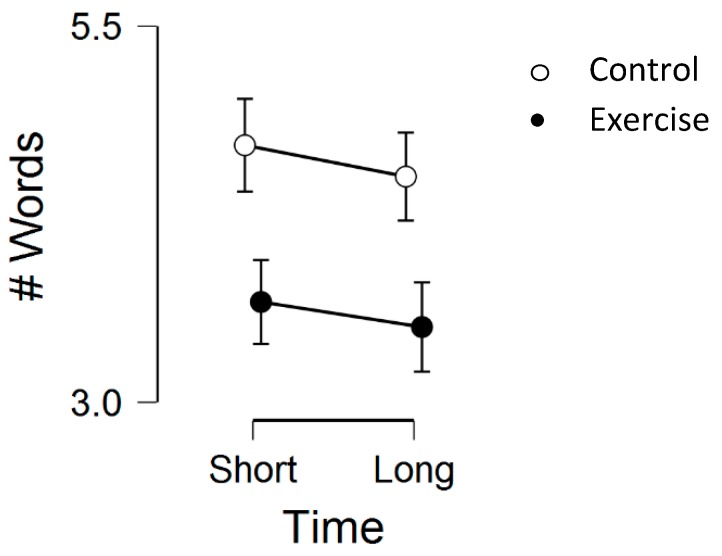
Episodic memory performance across the two conditions (exercise vs. control) and two time-points (short-term and long-term memory). Error bars represent standard errors.

There was a statistically significant main effect for condition, *F*(1, 23) = 4.50, *p* = 0.04, η^2^_p_ = 0.16, but no main effect for time, *F*(1, 23) = 1.95, *p* = 0.18, η^2^_p_ = 0.07, or time by condition interaction, *F*(1, 23) = 0.04, *p* = 0.84, η^2^_p_ = 0.002. Regarding condition effects, Bonferroni-corrected post-hoc tests indicated that the control group had a higher episodic memory performance than the exercise condition, *M*_diff_ = 1.02, *p* = 0.04, d = 0.43. See Figure 2 below for the episodic memory scores across the two conditions and 2 time-points.

## 4. Discussion—Experiment 1

In direct alignment with the transient hypofrontality hypothesis, high-intensity acute exercise was associated with reduced working memory and episodic memory function. This is the first experiment, to our knowledge, to evaluate this paradigm (transient hypofrontality) for both working memory and episodic memory within the context of acute exercise. As previously noted, the prefrontal cortex plays a critical role in working memory capacity [3]. As such, and per the transient hypofrontality hypothesis, we expected working memory to be reduced during an acute bout of high-intensity exercise.

Increased performance for episodic memory within this paradigm is plausible for several reasons. First, the amplitude and frequency of theta and gamma oscillations is positively correlated with the speed of running in rodents [36,37,38]. As such, high-intensity acute exercise increases hippocampal neural activity, likely priming neurons to integrate into the memory engram [39]. Second, episodic memory function, particularly a paired-associative episodic memory task, is heavily dependent upon the hippocampus [40]. Importantly, however, hippocampal-dependent paired-associative learning appears to be influenced more by scene imagery as opposed to item-binding [41]. We note that our episodic memory task utilized pairs of words within the middle range of imagery (i.e., 414–486, with imagery ratings ranging from 100–700). As such, future work should revisit this paradigm and utilize word pairs with a high degree of imagery, to better ensure greater activation of the hippocampus during paired-associative learning. Our finding that episodic memory was reduced during high-intensity acute exercise is not, however, entirely surprising given the role that the prefrontal cortex plays in episodic memory [5]. For example, previous work demonstrates that the left prefrontal cortex plays an important role in episodic memory encoding, whereas the right prefrontal cortex may help to facilitate memory retrieval [42].

Based on our findings that high-intensity acute exercise reduced memory function (working and episodic), Experiment 2 evaluated whether this effect was dependent on exercise intensity. That is, Experiment 2 was designed to replicate the findings from Experiment 1 (i.e., is memory reduced during high-intensity acute exercise?) as well as evaluate whether there is an intensity-specific effect.

## 5. Introduction—Experiment 2

It is plausible that a certain threshold of velocity of movement may be needed to induce this transient hypofrontality effect. As reviewed elsewhere [13], acute mild- and moderate-intensity exercise increase global cerebral blood flow, whereas for high-intensity exercise, cerebral blood flow returns to baseline levels. Specifically, for the right prefrontal cortex, however, oxyhemoglobin levels decrease below baseline when engaging in exercise at or greater than 80% of peak VO_2_ [43]. As such, we hypothesized that memory function during high-intensity acute exercise would, again, be reduced, but would not be reduced for low- to moderate-intensity exercise (i.e., below 80% of max). Thus, for Experiment 2, we evaluated memory performance at low (30%), moderate (50%), and high-intensity (80%) acute exercise.

## 6. Methods—Experiment 2

### 6.1. Study Design

A within-subject randomized controlled intervention was employed. Participants completed four visits, in a counterbalanced order. One visit involved a control visit, whereas the other three visits involved an acute bout of exercise at varying intensities. All visits occurred around the same time of day and within 48–72 h of each other. Participants provided written consent prior to participation. This study was approved by the ethics committee at the University of Mississippi.

### 6.2. Protocol for Visits

Participants (*n* = 24) completed four visits, including (1) memory function while seated (control), (2) memory function during acute exercise at 30% of HRR (light-intensity), (3) memory function during acute exercise at 50% of HRR (moderate-intensity), and (4) memory function during acute exercise at 80% of HRR (vigorous-intensity). Details for these visits are as follows (Table 5).

### 6.3. Participants

The sample included 24 young adults. The sampling approach and eligibility criteria for Experiment 2 were the same as Experiment 1.

### 6.4. Memory Outcome

The outcome measure was working memory capacity, as determined by the Brown–Peterson task. The same task and approach used in Experiment 1 was employed in Experiment 2.

### 6.5. Statistical Analysis

All statistical analyses were computed in JASP (v. 0.9.2.0). A two-factor repeated-measures ANOVA was computed. Specifically, a 4 (conditions) × 4 (delay period of 0, 9, 18, 36 s) repeated-measures ANOVA was utilized. Statistical significance was set at an alpha of 0.05. Partial eta-squared (η^2^_p_) was calculated as an effect size estimate for the ANOVA models, whereas Cohen’s *d* was calculated as an effect size estimate for the post-hoc analyses.

## 7. Results—Experiment 2

Table 6 displays the characteristics of the sample. Participants, on average, were 20.9 years of age and were predominately female (66.7%) and non-Hispanic white (83.3%).

Table 7 displays the heart rate responses to the exercise visits. There was a significant main effect for time, *F*(2, 46) = 1115.3, *p* < 0.001, η^2^_p_ = 0.98, condition, *F*(3, 69) = 280.4, *p* < 0.001, η^2^_p_ = 0.92, and time by condition interaction, *F*(6, 138) = 261.4, *p* < 0.001, η^2^_p_ = 0.92.

**Figure 3:**
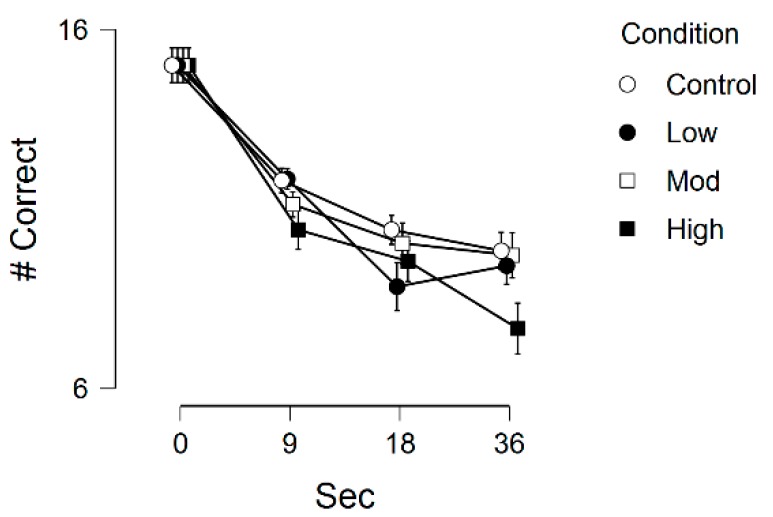
Working memory performance across the 4 conditions (control, low-intensity, moderate-intensity and high-intensity) and four time-points (0-sec, 9-sec, 18-sec, and 36-sec). Error bars represent standard errors.

Table 8 displays the memory outcomes across the experimental conditions, with Figure 3 showing these results schematically. There was a statistically significant main effect for time, *F*(3, 69) = 50.35, *p* < 0.001, η^2^_p_ = 0.69, main effect for condition, *F*(3, 69) = 3.30, *p* = 0.02, η^2^_p_ = 0.13, and significant time by condition interaction, *F*(9, 207) = 2.09, *p* = 0.03, η^2^_p_ = 0.08.

Regarding the time effects, Bonferroni-corrected post-hoc tests indicated that the 0-sec condition had greater working memory than the 9-sec period, *M*_diff_ = 3.70, *p* < 0.001, d = 1.49, 18-sec period, *M*_diff_ = 5.29, *p* < 0.001, d = 1.60, and 36-sec period, *M*_diff_ = 5.84, *p* < 0.001, d = 1.56. Similarly, the 9-sec period was different than the 18-sec period, *M*_diff_ = 1.58, *p* < 0.001, d = 0.94, and 36-sec period, *M*_diff_ = 2.13, *p* < 0.001, d = 1.17. Regarding the condition effects, working memory during the control condition was significantly better than the vigorous-intensity condition, *M*_diff_ = 1.10, *p* = 0.05, d = 0.58. There were no significant differences among any other combination across the conditions.

## 8. Discussion—Experiment 2

Experiment 2 provides evidence that working memory function is reduced during high-intensity acute exercise. Thus, Experiment 2 replicates the findings from Experiment 1. Further, results from Experiment 2 suggest that, when compared to a resting state of cognition, work memory function is not different during low- and moderate-intensity acute exercise.

Experiment 2 did not measure prefrontal cortex activation, but our results may, in part, be due to increased prefrontal cortex activation during low- and moderate-intensity exercise [13,43]. In alignment with this, Tsujii et al. [44] demonstrated that an acute bout of moderate-intensity exercise improved working memory performance and also increased prefrontal cortex activation during the memory task. Similarly, Yamazaki et al. [45] demonstrated than an acute bout of low-intensity exercise improved working memory performance, and among those classified as exercise responders, the acute bout of exercise also increased oxyhemoglobin levels in the right ventrolateral prefrontal cortex during the working memory task. Notably, however, in our study, for the 18-sec interval, working memory was numerically lower in the light-intensity condition when compared to the other conditions. Although other research has demonstrated that low-intensity exercise is associated with improved memory performance, perhaps in our sample, this intensity was too low to elicit improvements in working memory.

Experiment 2 was limited to a working memory task, as, due to logistical reasons (i.e., this would have required 8 total visits to the lab), we did not evaluate whether there is an intensity-specific effect of acute exercise on episodic memory function. This would be worth evaluating in the future. Similar to Experiment 1, for Experiment 2, the sample, which was relatively small, was predominately female. Past research suggests that females tend to perform better than males on various episodic memory tasks [46]. However, there is less evidence to suggest a sex-specific effect of acute exercise on memory function [47].

## 9. General Discussion

Experiment 1 demonstrates that both working memory and episodic memory are reduced during high-intensity acute exercise. Experiment 2 replicates this reduced performance effect of high-intensity acute exercise on working memory performance. Experiment 2 also demonstrates that, although working memory is reduced during high-intensity exercise, such an effect did not consistently occur for low- and moderate-intensity exercise. Future work on this topic should evaluate PFC oxygenation across the conditions to help provide mechanistic evidence of this transient hypofrontality paradigm. Such work should also consider evaluating psychological-based mechanisms, including, for example, exercise-based intensity-specific differences in psychological attention.

Our findings should not be confused with past findings suggesting, for example, that high-intensity acute exercise can improve both working memory and episodic memory function [6,7,8]. These studies employed the bout of acute exercise shortly before the memory task. We have thoroughly discussed these beneficial effects elsewhere [9].

Practical implications of this body of research are as follows. Acute exercise, even high-intensity exercise, can enhance working memory and episodic memory performance. Importantly, individuals may need to take a short recovery period after this bout of exercise before starting the learning session. Some individuals prefer to move (walk, stationary cycle) while learning/studying, and in such situations, our findings suggest that individuals should consider limiting the exercise intensity to low or moderate intensity. A sensible suggestion would also be to, for episodic memory, match the context during encoding and memory retrieval [48].

## 10. Conclusions

In conclusion, our experiments provide support for the transient hypofrontality hypothesis. Both working memory and episodic memory are impaired during high-intensity acute exercise. Working memory does not appear to be consistently reduced during lower exercise intensities.

## Figures and Tables

**Table 1:** Study protocol.

Visit	Start → → → → → → Finish							
**Control WM**		5-min seated rest	Complete WM task while seated	Leave lab				
**Exercise WM**	20-min of Exercise	5-min into the exercise bout, start WM task	WM task is finished prior to the end of the exercise bout	Leave lab				
**Control EPI**		5-min seated rest	Memory encoding while seated	30-sec of arithmetic problems while seated	Short-term cued-recall while seated	20-min of arithmetic problems while seated	Long-term cued-recall while seated	Leave lab
**Exercise EPI**	20-min of Exercise	5-min into the exercise bout, start EPI task		30-sec of arithmetic problems	Short-term cued-recall while exercising	Continuing exercising while completing simple arithmetic problems (verbal responding)	Long-term cued-recall (at exactly 20-min after short-term recall) while exercising	Leave lab

EPI, Episodic Memory; WM, Working Memory.

**Table 2:** Characteristics of the sample.

Variable	Mean (SD)
Age, mean years	21.9 (1.9)
Gender, % Female	70.8
Race-Ethnicity, % White	95.8
BMI, mean kg/m^2^	25.1 (3.6)
MVPA, mean min/week	142.9 (104.2)

MVPA, Moderate to vigorous physical activity (self-reported).

**Table 3:** Heart rate responses to the experimental manipulation.

Condition	Mean BPM
**Working Memory**	
*Control*	
Rest	77.9 (12.4)
Endpoint	73.4 (9.3)
*Exercise*	
Rest	82.6 (14.9)
Midpoint	157.8 (4.9)
Endpoint	158.3 (4.9)
**Episodic Memory**	
*Control*	
Rest	77.2 (12.7)
Endpoint	70.3 (9.5)
*Exercise*	
Rest	78.8 (9.2)
Midpoint	155.4 (5.0)
Endpoint	154.6 (5.0)

BPM, beats per minute.

**Table 4:** Displays the memory outcomes across the experimental conditions.

Memory	Exercise		Control	
**Working Memory**	**Mean**	**SD**	**Mean**	**SD**
0-sec	15.00	0.00	14.88	0.61
9-sec	11.96	3.32	13.00	2.22
18-sec	10.96	3.22	12.75	10.96
36-sec	10.75	3.41	11.67	2.79
**Episodic Memory**				
Short-term	3.67	2.88	4.71	3.23
Long-term	3.50	2.84	4.50	3.06

**Table 5:** Study protocol.

Visit	Start → → → → → Finish			
**Control**	5-min seated rest	Complete WM task while seated	Leave lab	
**Light**	Start acute bout of exercise (30% of HRR)	10-min into the exercise bout, start WM task	WM task is finished prior to the end of the exercise bout	Leave lab
**Moderate**	Start acute bout of exercise (50% of HRR)	10-min into the exercise bout, start WM task	WM task is finished prior to the end of the exercise bout	Leave lab
**Vigorous**	Start acute bout of exercise (80% of HRR)	10-min into the exercise bout, start WM task	WM task is finished prior to the end of the exercise bout	Leave lab

WM, Working Memory.

**Table 6:** Characteristics of the sample.

Variable	Mean (SD)
Age, mean years	20.9 (1.1)
Gender, % Female	66.7
Race-Ethnicity, % White	83.3
BMI, mean kg/m^2^	24.3 (3.6)
MVPA, mean min/week	167.1 (130.5)

**Table 7:** Heart rate responses to the exercise manipulation.

Condition	Mean (SD) BPM
*Control*	
Rest	75.7 (12.7)
Midpoint	76.6 (12.1)
Endpoint	76.7 (12.9)
*Low-Intensity*	
Rest	74.2 (10.5)
Midpoint	110.6 (9.2)
Endpoint	111.6 (7.6)
*Moderate-Intensity*	
Rest	74.5 (10.4)
Midpoint	132.3 (7.6)
Endpoint	131.4 (7.6)
*High-Intensity*	
Rest	75.0 (11.5)
Midpoint	161.2 (9.0)
Endpoint	163.8 (6.7)

BPM, beats per minute.

**Table 8:** Working memory results across the exercise conditions.

Working Memory	Control	Light-Intensity	Moderate-Intensity	High-Intensity
0-sec	15.0 (0.0)	15.0 (0.0)	15.0 (0.0)	15.0 (0.0)
9-sec	11.79 (2.4)	11.83 (2.8)	11.12 (3.1)	10.41 (3.4)
18-sec	10.41 (3.5)	8.83 (4.3)	10.04 (4.0)	9.54 (4.24)
36-sec	9.83 (3.9)	9.41 (3.9)	9.70 (4.5)	7.66 (5.1)
